# DNA Barcode Identification of Podocarpaceae—The Second Largest Conifer Family

**DOI:** 10.1371/journal.pone.0081008

**Published:** 2013-11-27

**Authors:** Damon P. Little, Patrick Knopf, Christian Schulz

**Affiliations:** 1 Lewis B. and Dorothy Cullman Program for Molecular Systematics, The New York Botanical Garden, Bronx, New York, United States of America; 2 Lehrstuhl für Evolution und Biodiversität der Pflanzen, Ruhr–Universität Bochum, Bochum, Nordrhein–Westfalen, Bundesrepublik Deutschland; University of Guelph, Canada

## Abstract

We have generated *matK*, *rbcL*, and nrITS2 DNA barcodes for 320 specimens representing all 18 extant genera of the conifer family Podocarpaceae. The sample includes 145 of the 198 recognized species. Comparative analyses of sequence quality and species discrimination were conducted on the 159 individuals from which all three markers were recovered (representing 15 genera and 97 species). The vast majority of sequences were of high quality (*B*_30_ = 0.596–0.989). Even the lowest quality sequences exceeded the minimum requirements of the BARCODE data standard. In the few instances that low quality sequences were generated, the responsible mechanism could not be discerned. There were no statistically significant differences in the discriminatory power of markers or marker combinations (*p* = 0.05). The discriminatory power of the barcode markers individually and in combination is low (56.7% of species at maximum). In some instances, species discrimination failed in spite of ostensibly useful variation being present (genotypes were shared among species), but in many cases there was simply an absence of sequence variation. Barcode gaps (maximum intraspecific p–distance > minimum interspecific p–distance) were observed in 50.5% of species when all three markers were considered simultaneously. The presence of a barcode gap was not predictive of discrimination success (*p* = 0.02) and there was no statistically significant difference in the frequency of barcode gaps among markers (*p* = 0.05). In addition, there was no correlation between number of individuals sampled per species and the presence of a barcode gap (*p* = 0.27).

## Introduction

Podocarpaceae is a family of evergreen trees and shrubs that are sometimes cultivated as ornamentals in suitably warm climates. In terms of number of species, Podocarpaceae is the second largest family of conifers [1]. Podocarpaceae are often a minor subcanopy component of angiosperm–dominated forests. They are most abundant in the mid– to high–elevation tropics where they thrive on nutrient–poor soils. In addition, Podocarpaceae are found in some unusual low–elevation forest types (e.g. kerangas of Borneo; [2]).

Accurate identification of tropical forest trees, such as Podocarpaceae, is often very difficult. The most easily accessed material is usually sterile. If fertile material is present, it is frequently either inaccessible or detached from the tree making it difficult to convincingly associate the fertile and sterile portions. Although sterile material of Podocarpaceae can usually be identified to genus using phyllotaxis and leaf form [3], [4], accurate species identification often requires careful microscopic examination of internal [5]–[20] and external characteristics [21]–[27]. Proper use of the existing identification tools requires training in botanical terminology, skill in microtechnique, and familiarity with Podocarpaceae.

Species of Podocarpaceae are of conservation concern primarily as a result of small population sizes and limited available habitat. Twenty–seven Podocarpaceae species are included in the International Union for the Conservation of Nature (IUCN; [28]) red list under the categories of vulnerable (10 species), endangered (14 species), and critically endangered (three species). Two species are included in the appendices of the Convention on International Trade in Endangered Species (CITES; [29]): *Podocarpus parlatorei* is listed in Appendix I (trade is not allowed) and *Po. neriifolius* is listed in Appendix III (trade with, some limitations, is allowed).

Podocarpaceae have a minor role in commerce. *Nageia nagi*, when labeled as Asian bayberry, can legally be sold in the United States of America as an herbal dietary supplement [30]. The seeds are processed into an edible oil that is also used in manufacturing [31]. The young leaves are also edible, but not typically consumed [32]. The conspicuous fleshy reproductive structures (receptacles or epimatium) of *Afrocarpus falcatus*, *Dacrycarpus dacrydioides*, *Dacrydium cupressinum*, *Po. elatus*, *Po. macrophyllus*, *Po. totara*, and *Prumnopitys taxifolia* are eaten either raw or cooked [32].

Although their use is currently very limited, Podocarpaceae are known to have medicinal properties that benefit humans and animals [33], [34]. The receptacles and leaves contain a variety of bio–active compounds such as antioxidants, nordi–terpenes, podocarpic acid, and tatarol [33], [35], [36]. Some of these compounds have antimicrobial, fungistatic, or bacteriostatic properties [33], [37], [38]. Other compounds have cytotoxic properties that may be useful in destroying cancer [39]–[43].

The rarity of large uniform stands coupled with a slow rate of growth for most species makes harvest of Podocarpaceae wood generally unsustainable [2]. The growth rate of *Po. totara* may however accommodate sustainable harvest [44]. Relative scarcity results in a meager international trade—primarily originating from New Zealand and South Africa [2], [45]–[47]. Timber from Podocarpaceae, referred to as ‘podo’ (or ‘yellow yew’) in commerce, has straight even grain. The wood of some species is brittle when worked and not particularly durable outdoors [46], [47]. Wood from *Po. totara* is durable and highly amenable to industrial machining [44]. Wood from *Lepidothamnus intermedius*, *Manoao colensoi*, and *Pr. taxifolia* is very rot resistant [48]. Timber of *Ma. colensoi* and *Le. intermedius* have long been used for railway ties [48]. In addition, the wood of some Podocarpaceae species is highly insect resistant (e.g. *Af. gracilior* [49], *Po. hallii* [50], *Po. macrophyllus* [51], and *Po. nivalis* [50]).

A reference library of Podocarpaceae DNA barcodes will allow researchers unfamiliar with the family's morphology and anatomy to make accurate identifications. We hope that DNA barcodes will permit foresters, ecologists, conservationists, customs authorities, etc. to make accurate biodiversity inventories and to monitor trade in threatened and endangered Podocarpaceae species so that future conservation and management decisions can be based on sound data.

We aim to generate and evaluate a DNA barcode reference library for Podocarpaceae. The library will be assessed both by the quality of the constituent sequences and the degree to which observed sequence variation unambiguously distinguishes Podocarpaceae species from one another.

## Materials and Methods

We sampled 320 individuals representing all of the 18 extant genera of Podocarpaceae (including *Phyllocladus*). Our sample included 145 of the 198 recognized species (73.2%; [1]). Between 1 and 9 individuals per species were sequenced (median  = 2; IQR  = 1–3). All samples were expert–identified using a combination of morphology and leaf anatomy [20]. Voucher information is in Dataset S1.

### Molecular techniques

DNA was extracted from herbarium specimens or silica–dried tissue using the Qiagen DNeasy96 kit. The manufacturer's protocol was modified for herbarium specimens: instead of the recommended incubation, homogenized tissue was digested with 30 µL (20 µg/µL stock) of proteinase K in 400 µL AP1 (supplied by the manufacturer) and 1 µL of DX (supplied by the manufacturer) at 42°C for 24 hours with slow mixing (60 rotations per minute).

The Polymerase Chain Reaction (PCR) was used to amplify *matK* in a 15 µL volume containing: 20 mM tris pH 8.8, 10 mM KCl, 10 mM (NH_4_)_2_ SO_4_, 2 mM MgSO_4_, 0.1% (v/v) Triton X-100, 5% (w/v) sucrose, 0.025% (w/v) cresol red, 0.025 µg/µL BSA, 0.2 mM dNTPs, 1 µM of Gym_F1A (5′-ATY GYR CTT TTA TGT TTA CAR GC-3′; [53]), 1 µM of Gym_R1A (5′-TCA YCC GGA RAT TTT GGT TCG-3′; [53]), 0.5 units *Taq*, and 0.5 µL genomic DNA. The reaction mixture was incubated for 150 sec at 95°C, cycled 35 times (30 sec at 95°C, 60 sec at 52°C, 40 sec at 72°C) and then incubated at 72°C for 10 minutes.

PCR amplification of nrITS2 was similar to that of *matK* except primer annealing was carried out for 30 sec at 58°C rather than 60 sec at 52°C and primers S2F (5′-ATG CGA TAC TTG GTG TGA AT-3′; [53]) and S3R (5′-GAC GCT TCT CCA GAC TAC AAT-3′ [53]) replaced Gym_F1A and Gym_R1A.

Unused primers and dNTPs were neutralized using ExoSAP-IT (USB). PCR products were bidirectionally sequenced, using the amplification primers, with BigDye v3.1 (Life Technologies) at the High–Throughput Genomics Unit (University of Washington). PCR amplification and sequencing of *rbcL* was described previously [54].

### Data analysis

Bases were called and quality values (QV) assigned using KB 1.4 (Life Technologies). Sequencer 4.1 (Gene Codes) was used to construct sequence contigs, trim contigs to a uniform beginning/end (priming sites were excluded), and resolve differences between sequencing reads. Sequences of *matK* and *rbcL* were checked for stop codons and frameshift mutations. To identify potential contaminates, all sequences were queried against GenBank using BLAST 2.2.26 [55]. Only hits with an e–value of 10^−20.0^ or less were retained. Additional contaminates were identified by aligning each marker with MUSCLE 3.8 [56], coding the resulting indels using ‘simple indel coding’ [57], [58], and resampling the resulting matrix 1000 times using the jackknife [59] procedure. For each resampled matrix, the search for optimal trees was conducted in TNT 1.1 [60]. The search consisted of ten random addition replicates with five trees held in memory per replicate and SPR followed by TBR (BB) branch swapping. The strict consensus tree from each resampled matrix was used to calculate the jackknife tree.

In order to make meaningful comparisons across makers, we only analyzed data from specimens for which *matK*, *rbcL*, and nrITS2 were able to be sequenced—referred to as ‘complete samples’ hereafter. Sequences from specimens that could not be definitively identified using morphology/anatomy were excluded.

Sequence quality was assessed using the barcode quality index (*B*; [61]) with the acceptable quality threshold (*q*) set to 30 (an average of one error per thousand sequenced bases). The expected coverage (*x*) was set to 2. The contig size (*c*) was set to the observed size. Linguistic complexity (*LC*; [62]), a measure of sequence repetitiveness, was calculated for each sequence, using COMPLEX 6.1.0 [63] with window size set to 100 bases, step size set to 1 base, minimum pattern size set to 3 bases, and maximum pattern size set to 6 bases. The threshold for significant increase in homopolymer (mononucleotide repeats) induced PCR artifacts has been empirically determined to be eight bases [64]—thus sequences with homopolymers, eight bases or longer, were identified. Statistical differences, in sequence quality, linguistic complexity, and homopolymer frequency among markers were evaluated with Scheffé's test [65]–[67] at *p* = 0.05 using the Gaussian distribution. Correlations between sequence quality and linguistic complexity as well as sequence quality and homopolymer frequency were measured by Spearman's rank correlation tests [67], [68].

TNT 1.1 [60] was used to analyze phylogenetic relationships among complete samples. Each marker was aligned with MUSCLE 3.8 [56], indels were coded using ‘simple indel coding’ [57], [59], and markers were combined by concatenation [69]. The resulting matrix was searched for optimal trees using 1000 random addition replicates: for each replicate two trees were held in memory, SPR branch swapping was followed by TBR (BB) branch swapping and a 200 iteration ratchet [70] perturbing 8% of the characters per iteration (4% up weighted, 4% down weighted). Clade support was assessed by 10,000 jackknife resamplings [59]. For each resampled matrix, the search for optimal trees consisted of ten random addition replicates with five trees held in memory per replicate and SPR followed by TBR (BB) branch swapping. The jackknife frequency of each clade in the strict consensus of the original matrix was calculated with SUMTREES 3.3.1 [71] using the strict consensus tree from each resampled matrix [67], [72]. Trees were rooted following [54]. Tree–based species discrimination was assessed using the ‘least inclusive clade’ method [73].

Species discrimination was calculated using BRONX 2.0 [74], [75]. Discrimination success would be overestimated if the reference database just included sequences in the complete sample—thus a BRONX reference database was constructed from all sequences for each marker and marker combination (Dataset S1). To calculate species discrimination, sequences of each complete sample were queried against the reference database. Species were considered distinct if all queries for a given species returned only sequences belonging to that species. The binomial distribution, with each species considered an independent test, was used to compute 95% confidence intervals [67], [76], [77]. Differences in species discrimination among markers and marker combinations were quantified using Scheffé's test [65]–[67] at *p* = 0.05. The binomial distribution was used for tests of species discrimination and the Gaussian distribution was used to test if the number of species conflated when identification failed varied among markers.

Relative variation within and among species—the ‘barcode gap’ [78]—was quantified by comparing pairwise distances for complete samples. Each pair of sequences in the complete sample was aligned separately with MUSCLE 3.8 [56] and the number of unambiguous nucleotide differences was divided by the total number of aligned positions to calculate the edit distance (uncorrected p–distance; [79]). To minimize sampling and analytic artifacts, the maximum intraspecific distance was compared to the minimum interspecific distance for each species [80]. For each marker, the frequency of barcode gap (maximum intraspecific > minimum interspecific) occurrence was assessed using the binomial distribution and Scheffé's test [65]–[67] at *p* = 0.05. The point–biserial correlation coefficient was used to examine the relationship between number of samples per species and the occurrence of a barcode gap [67], [81]. Sequences from all three markers were used simultaneously with McNemar's test [67], [82] to measure the correlation between the occurrence of a barcode gap and whether or not a species can be consistently distinguished from all other species using diagnostic nucleotide positions.

## Results and Discussion

**Figure 1:**
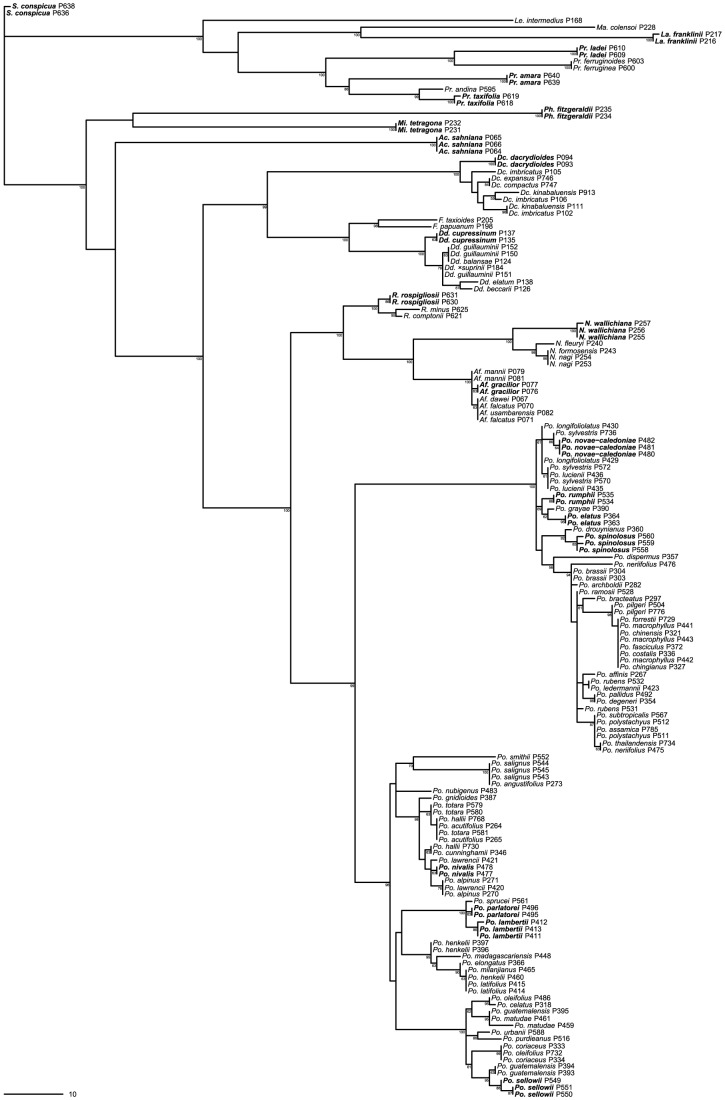
Phylogenetic relationships among complete samples. Strict consensus of 3600 most parsimonious trees (L = 1205; CI = 0.59; RI = 0.93; all tree statistics exclude uninformative nucleotide positions) obtained from the simultaneous analysis of *matK*, *rbcL*, and nrITS2 sequence data. Numbers at nodes indicated jackknife support above 50%. Species that can be distinguished from all other species using the ‘least inclusive clade’ method are in boldface (the least inclusive clade method cannot be applied to species with only one sample). Genera have been abbreviated: *Ac.*  =  *Acmopyle*, *Af.*  =  *Afrocarpus*, *Dc.*  =  *Dacrycarpus*, *Dd.*  =  *Dacrydium*, *F.*  =  *Falcatifolium*, *La.*  =  *Lagarostrobos*, *Le.*  =  *Lepidothamnus*, *Ma.*  =  *Manoao*, *Mi.*  =  *Microcachrys*, *N.*  =  *Nageia*, *Ph.*  =  *Pherosphaera*, *Po.*  =  *Podocarpus*, *Pr.*  =  *Prumnopitys*, *R.*  =  *Retrophyllum*, and *S.*  =  *Saxegothaea*. Sample codes correspond to those used in Dataset S1.

In total, 281 *matK*, 202 *rbcL*, and 212 nrITS2 finished sequences were generated (Dataset S1). BLAST [55] queries indicate that the newly generated sequences are consistent with other samples of Podocarpaceae deposited in GenBank (data not shown). Phylogenetic arrangement of genera and species is roughly consistent with previous molecular phylogenetic studies (Figure 1; [54], [83]–[86]). Sequences derived from individuals of the same species are always in close phylogenetic proximity, but in some cases the sequences do not form a monophyletic group (sensu [87]). Sequences of some morphological/anatomical species are unambiguously polyphyletic (sensu [87]; e.g. *Podocarpus oleifolius*, Figure 1). Mismatches between morphological/anatomical species circumscription and barcode sequences warrant further investigation as they may indicate the presence of cryptic species, introgression, or ancestral polymorphism followed by incomplete lineage sorting. Together the BLAST and phylogenetic contaminate screens indicate that the sequences generated are indeed Podocarpaceae and that no PCR artifacts or errors in sample handling could be detected.

Newly generated sequences of *matK* vary from 760 to 775 bp (median  = 769; IQR  = 769–769), sequences of *rbcL* are uniformly 607 bp, and sequences of nrITS2 vary from 420 to 435 bp (median  = 425; IQR  = 425–425). The multiple sequence alignment of *matK* results in six indels—3 bp (two indels), 6 bp (three indels), and 9 bp (one indel), respectively. The 20 nrITS2 indels resulting from multiple sequence alignment range from 1 to 17 bp (median  = 1; IQR  = 1–2).

Of the 320 individuals sequenced, finished *matK*, *rbcL*, and nrITS2 sequences were generated for 159 individuals. These samples, representing 15 of the 18 extant genera (83.3%; [1]; *Halocarpus*, *Parasitaxus*, and *Phyllocladus* are not included in the complete sample) and 97 of the 198 recognized species (48.9%; [1]), were analyzed for sequence quality, linguistic complexity, species discrimination, and barcode gaps. The complete sample set contained between 1 and 3 individuals per species (median  = 1; IQR  = 1–2; Table 1). In total, there were 95 distinct *matK* sequence types, 70 *rbcL* sequence types, and 81 nrITS2 sequence types. The complete sample contained 71 (74.7%) *matK* sequence types, 61 (87.1%) *rbcL* sequence types, and 65 (80.2%) nrITS2 sequence types for a combined 90 distinct multilocus genotypes.

**Table 1:** Diagnostic barcode variation for complete samples of Podocarpaceae.

species	*n*	discriminatory success				multilocus barcode gap	most frequently conflated species (ordered by frequency)
		*matK*	*rbcL*	nrITS2	combined		
*Ac. sahniana*	3	+	+	+	+	1	—
*Af. dawei*	1	−	−	−	−	0	*Af. gracilior*, *Af. mannii*, *Af. falcatus*, et al.
*Af. falcatus*	2	−	−	−	−	0	*Af. gracilior*, *Af. mannii*, *Af. usambarensis*, et al.
*Af. gracilior*	2	−	−	+	+	0	*Af. falcatus*, *Af. usambarensis*, *Af. dawei*, et al.
*Af. mannii*	2	−	−	−	+	0	*Af. falcatus*, *Af. usambarensis*, *Af. dawei*, et al.
*Af. usambarensis*	1	−	−	−	−	0	*Af. gracilior*, *Af. mannii*, *Af. falcatus*, et al.
*Dc. compactus*	1	−	−	−	−	0	*Dc. kinabaluensis*, *Dc. imbricatus*, *Dc. expansus*
*Dc. dacrydioides*	2	+	+	+	+	1	—
*Dc. expansus*	1	−	−	−	−	0	*Dc. kinabaluensis*, *Dc. imbricatus*, *Dc. compactus*
*Dc. imbricatus*	3	−	−	+	+	0	*Dc. expansus*, *Dc. compactus*, *Dc. kinabaluensis*
*Dc. kinabaluensis*	2	−	−	−	+	0	*Dc. expansus*, *Dc. compactus*, *Dc. imbricatus*
*Dd. balansae*	1	−	−	−	−	0	*Dd. araucarioides*, *Dd. cupressinum*, *Dd. nausoriense*, et al.
*Dd. beccarii*	1	−	+	+	+	1	*Dd. gracile*, *Dd. xanthandrum*
*Dd. cupressinum*	2	+	−	+	+	1	*Dd. guillauminii*, *Dd. araucarioides*, *Dd. balansae*, et al.
*Dd. elatum*	1	+	+	+	+	1	—
*Dd. guillauminii*	3	−	−	−	−	0	*Dd. araucarioides*, *Dd. cupressinum*, *Dd. nausoriense*, et al.
*Dd. ×suprinii*	1	−	−	−	−	0	*Dd. araucarioides*, *Dd. cupressinum*, *Dd. nausoriense*, et al.
*F. papuanum*	1	+	+	+	+	1	—
*F. taxioides*	1	+	+	+	+	1	—
*La. franklinii*	2	+	+	+	+	1	—
*Le. intermedius*	1	+	+	+	+	1	—
*Ma. colensoi*	1	+	−	+	+	1	*Le. intermedius*
*Mi. tetragona*	2	+	+	+	+	1	—
*N. fleuryi*	1	+	+	+	+	1	—
*N. formosensis*	1	−	−	−	−	0	*N. motleyi*, *N. nagi*
*N. nagi*	2	−	−	−	−	0	*N. motleyi*, *N. formosensis*
*N. wallichiana*	3	+	−	+	+	1	*N. motleyi*
*Ph. fitzgeraldii*	2	+	+	+	+	1	—
*Po. acutifolius*	2	−	−	−	−	0	*Po. cunninghamii*, *Po. totara*, *Po. hallii*
*Po. affinis*	1	−	+	+	+	1	*Po. degeneri*, *Po. rubens*, *Po. insularis*, et al.
*Po. alpinus*	2	−	−	−	−	0	*Po. lawrencei*, *Po. gnidioides*, *Po. nivalis*, et al.
*Po. angustifolius*	1	−	−	−	−	0	*Po. salignus*
*Po. archboldii*	1	+	−	−	+	1	*Po. drouynianus*, *Po. polystachyus*, *Po. thailandensis*, et al.
*Po. assamica*	1	−	−	−	−	0	*Po. drouynianus*, *Po. deflexus*, *Po. insularis*, et al.
*Po. bracteatus*	1	−	−	−	+	1	*Po. neriifolius*, *Po. pseudobracteatus*
*Po. brassii*	2	−	−	−	+	1	*Po. drouynianus*, *Po. thailandensis*, *Po. deflexus*, et al.
*Po. celatus*	1	−	−	−	+	1	*Po. guatemalensis*, *Po. coriaceus*, *Po. sellowii*, et al.
*Po. chinensis*	1	−	−	−	−	0	*Po. nakaii*, *Po. pilgeri*, *Po. annamiensis*, et al.
*Po. chingianus*	1	−	−	−	−	0	*Po. nakaii*, *Po. pilgeri*, *Po. annamiensis*, et al.
*Po. coriaceus*	2	−	−	−	−	0	*Po. guatemalensis*, *Po. trinitensis*, *Po. celatus*, et al.
*Po. costalis*	1	−	−	−	−	0	*Po. nakaii*, *Po. pilgeri*, *Po. annamiensis*, et al.
*Po. cunninghamii*	1	−	−	−	−	0	*Po. totara*, *Po. acutifolius*, *Po. hallii*
*Po. degeneri*	1	−	−	−	−	0	*Po. affinis*, *Po. insularis*, *Po. ledermannii*, et al.
*Po. dispermus*	1	+	+	+	+	1	—
*Po. drouynianus*	1	−	+	−	+	1	*Po. polystachyus*, *Po. thailandensis*, *Po. insularis*, et al.
*Po. elatus*	2	+	+	+	+	1	—
*Po. elongatus*	1	+	+	−	+	1	*Po. polystachyus*, *Po. milanjianus*, *Po. henkelii*, et al.
*Po. fasciculus*	1	−	−	−	−	0	*Po. nakaii*, *Po. pilgeri*, *Po. annamiensis*, et al.
*Po. forrestii*	1	−	−	−	−	0	*Po. nakaii*, *Po. pilgeri*, *Po. annamiensis*, et al.
*Po. gnidioides*	1	+	−	+	+	1	*Po. alpinus*, *Po. lawrencii*
*Po. grayae*	1	+	−	+	+	1	*Po. lucienii*, *Po. rumphii*, *Po. sylvestris*, et al.
*Po. guatemalensis*	3	−	−	−	−	0	*Po. tepuiensis*, *Po. brasiliensis*, *Po. coriaceus*, et al.
*Po. hallii*	2	−	−	−	−	0	*Po. totara*, *Po. acutifolius*, *Po. cunninghamii*
*Po. henkelii*	3	−	−	−	−	0	*Po. polystachyus*, *Po. elongatus*, *Po. milanjianus*, et al.
*Po. lambertii*	3	+	+	+	+	1	—
*Po. latifolius*	2	−	−	−	−	0	*Po. polystachyus*, *Po. elongatus*, *Po. milanjianus*, et al.
*Po. lawrencii*	2	−	−	−	−	0	*Po. lawrencei*, *Po. gnidioides*, *Po. nivalis*, et al.
*Po. ledermannii*	1	−	−	−	−	0	*Po. polystachyus*, *Po. thailandensis*, *Po. decipiens*, et al.
*Po. longifoliolatus*	2	−	−	−	+	1	*Po. rumphii*, *Po. grayae*, *Po. lucienii*, et al.
*Po. lucienii*	2	−	−	−	−	0	*Po. rumphii*, *Po. grayae*, *Po. decumbens*, et al.
*Po. macrophyllus*	3	−	−	−	−	0	*Po. nakaii*, *Po. pilgeri*, *Po. annamiensis*, et al.
*Po. madagascariensis*	1	+	+	+	+	1	—
*Po. matudae*	2	−	−	−	−	0	*Po. oleifolius*, *Po. guatemalensis*
*Po. milanjianus*	1	−	−	−	−	0	*Po. polystachyus*, *Po. elongatus*, *Po. henkelii*, et al.
*Po. neriifolius*	2	−	−	−	−	0	*Po. drouynianus*, *Po. insularis*, *Po. ledermannii*, et al.
*Po. nivalis*	2	−	+	−	+	1	*Po. lawrencei*, *Po. totara*, *Po. acutifolius*, et al.
*Po. novae-caledoniae*	3	−	−	−	+	1	*Po. sylvestris*, *Po. beecherae*
*Po. nubigenus*	1	−	+	+	+	1	*Po. atjehensis*
*Po. oleifolius*	2	−	−	−	−	0	*Po. trinitensis*, *Po. salicifolius*, *Po. rusbyi*, et al.
*Po. pallidus*	1	−	−	−	−	0	*Po. affinis*, *Po. insularis*, *Po. ledermannii*, et al.
*Po. parlatorei*	2	−	−	+	+	1	*Po. transiens*, *Po. sprucei*
*Po. pilgeri*	2	−	+	−	+	1	*Po. nakaii*, *Po. costalis*, *Po. fasciculus*, et al.
*Po. polystachyus*	2	−	−	−	+	0	*Po. drouynianus*, *Po. deflexus*, *Po. insularis*, et al.
*Po. purdieanus*	1	+	+	+	+	1	—
*Po. ramosii*	1	−	−	−	+	1	*Po. thailandensis*, *Po. insularis*, *Po. assamica*, et al.
*Po. rubens*	2	−	−	−	−	0	*Po. drouynianus*, *Po. polystachyus*, *Po. thailandensis*, et al.
*Po. rumphii*	2	−	−	+	+	1	*Po. lucienii*, *Po. sylvestris*, *Po. grayae*, et al.
*Po. salignus*	3	−	−	−	−	0	*Po. angustifolius*
*Po. sellowii*	3	+	−	−	+	1	*Po. tepuiensis*, *Po. celatus*, *Po. oleifolius*, et al.
*Po. smithii*	1	+	+	+	+	1	—
*Po. spinolosus*	3	−	−	+	+	1	*Po. dispermus*, *Po. drouynianus*, *Po. rostratus*, et al.
*Po. sprucei*	1	−	−	−	+	1	*Po. glomeratus*, *Po. lambertii*, *Po. transiens*, et al.
*Po. subtropicalis*	1	−	−	−	−	0	*Po. drouynianus*, *Po. deflexus*, *Po. insularis*, et al.
*Po. sylvestris*	3	−	−	−	−	0	*Po. rumphii*, *Po. grayae*, *Po. beecherae*, et al.
*Po. thailandensis*	1	−	−	−	−	0	*Po. drouynianus*, *Po. insularis*, *Po. ledermannii*, et al.
*Po. totara*	3	−	−	−	−	0	*Po. cunninghamii*, *Po. acutifolius*, *Po. hallii*
*Po. urbanii*	1	+	−	+	+	1	*Po. guatemalensis*, *Po. celatus*, *Po. oleifolius*, et al.
*Pr. amara*	2	+	+	+	+	1	—
*Pr. andina*	1	+	−	+	+	1	*Pr. exigua*, *Pr. montana*
*Pr. ferruginea*	1	−	−	−	−	0	*Pr. ferruginoides*
*Pr. ferruginoides*	1	−	−	+	+	0	*Pr. ferruginea*
*Pr. ladei*	2	+	+	+	+	1	—
*Pr. taxifolia*	2	+	+	+	+	1	—
*R. comptonii*	1	+	−	−	+	1	*R. minus*, *R. rospigliosii*, *R. vitiense*
*R. minus*	1	−	−	+	+	1	*R. comptonii*
*R. rospigliosii*	2	+	+	−	+	1	*R. comptonii*, *R. vitiense*
*S. conspicua*	2	+	+	+	+	1	—

Genera have been abbreviated: Ac.  =  Acmopyle, Af.  =  Afrocarpus, Dc.  =  Dacrycarpus, Dd.  =  Dacrydium, F.  =  Falcatifolium, La.  =  Lagarostrobos, Le.  =  Lepidothamnus, Ma.  =  Manoao, Mi.  =  Microcachrys, N.  =  Nageia, Ph.  =  Pherosphaera, Po.  =  Podocarpus, Pr.  =  Prumnopitys, R.  =  Retrophyllum, and S.  =  Saxegothaea. Species that can be consistently distinguished from all other species are indicated by a ‘+’ while those that are conflated with other species are indicated with a ‘−’. The presence of a barcode gap is indicated with ‘1’, absence of a barcode gap with ‘0’.

### Sequence quality and complexity

**Figure 2:**
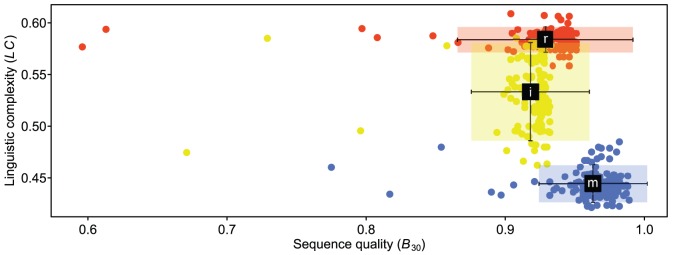
Barcode sequence quality (*B_30_*) versus linguistic complexity (*LC*) for complete samples. Circles represent individual *matK* (blue; m), *rbcL* (red; r), and nrITS2 (yellow; i) sequences. Black squares indicate marker means. Error bars span three standard deviations.

Sequence quality, as measured by *B*_30_ [61], ranged from 0.775 to 0.989 for *matK* (median  = 0.967; IQR  = 0.960–0.975), 0.596 to 0.951 for *rbcL* (median  = 0.938; IQR  = 0.929–0.944), and 0.671 to 0.933 for nrITS2 (median  = 0.924; IQR  = 0.919–0.927; Figure 2). The vast majority of sequences were of high quality: across all markers, 93.5% of the positions in the median sequence were assigned a quality value of 30 or greater—indicating that few, if any, of the finished sequences contain erroneous base calls. Although differences in sequence quality among markers was statistically significant (*p* = 0.05; *matK* > *rbcL* > nrITS2), even the lowest quality sequences exceeded the minimum requirements of the BARCODE data standard (version 2.3; [88])—thus the statistical differences observed are not particularly meaningful in practice.

Published *B*_30_ values for sequences generated using different primer sets are not directly comparable to those reported here because the primer sets define (slightly) different marker regions. Although not comparable in all cases, the median Podocarpaceae sequence is of higher quality than the average sequence reported for angiosperms across all three markers [61], [89], [90]. The largest comparable difference between literature reports and newly generated Podocarpaceae sequences was observed in nrITS2 (0.829 versus 0.924; [89]). The high quality of Podocarpaceae *matK* sequences is notable, but the gymnosperm specific primers [52] used to generate the *matK* sequences make direct comparisons to published values for angiosperms tenuous.

Linguistic complexity is a measure of the number of repeated ‘words’ in a sequence (words 3–6 bp were examined in this case; [62]). Sequences of *matK*, *rbcL*, and nrITS2 have statistically distinct linguistic complexity (*p* = 0.05) with *matK* being the simplest (median  = 0.443; IQR  = 0.437–0.449), followed by nrITS2 (median  = 0.527; IQR  = 0.513–0.566), and *rbcL* (median  = 0.584; IQR  = 0.577–0.590; Figure 2). The range of nrITS2 linguistic complexity is relatively broad especially in comparison to that of *matK* and *rbcL*—perhaps a result of different functional constraints on structural versus protein coding sequences.

One might expect that sequences with lower linguistic complexity (i.e. those with homopolymers and/or simple sequence repeats) will have lower sequence quality due to slip–strand mispairing at the site of repetitive sequence elements [64], [91]–[93], however lower linguistic complexity is correlated with higher sequence quality in Podocarpaceae (*p* <2.2×10^−16^). The sequences with the lowest linguistic complexity generally have sequence quality typical for the marker in question (Figure 2).

A homopolymer eight bases or longer was found in 35 sequences of the complete sample: 34 were *matK* sequences and one was an nrITS2 sequence. In the *matK* sequences from the complete sample, there is a single occurrence of A_8_ and 33 occurrences of T_8_. The T_8_ homopolymers occupy alignment positions 465–472 (found in all samples of *Afrocarpus*, *Lepidothamnus*, *Nageia*, *Prumnopitys*, and *Retrophyllum*) and 720–727 (found in some *Podocarpus*). The frequency of homopolymer occurrence was significantly different among markers (*p* = 0.05). Counter to previous findings [92], [93], high homopolymer frequency is correlated with high sequence quality in Podocarpaceae (*p* = 2.1×10^−16^). Previous investigations of the relationship between homopolymers and sequence quality focused on homopolymers ten bases or longer because they consistently result in low sequence quality [91]–[93], however homopolymers ten bases or longer are not found in any sequence of the complete sample. Thus we cannot determine if this length homopolymer has any effect on sequence quality.

The observed correlation between increased sequence quality and decreased linguistic complexity as well as the correlation between increased sequence quality and increased homopolymer frequency indicate that a mechanism other than slip–strand mispairing is responsible for the low quality sequences in the complete sample.

### Species discrimination

**Figure 3:**
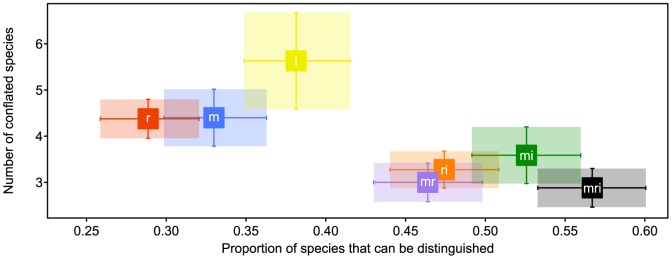
Species discrimination by barcode marker for complete samples. Squares indicate means for *matK* (blue; m), *rbcL* (red; r), nrITS2 (yellow; i), *matK* combined with *rbcL* (purple; mr), *matK* combined with nrITS2 (green; mi), *rbcL* combined with nrITS2 (orange, ri), and all markers combined (black; mri). Error bars indicate 95% confidence intervals.

For individual markers, BRONX [74], [75] species discrimination ranged from 28.8% to 38.1% (Figure 3; Table 1). Discrimination for marker combinations was slightly better at 46.4% to 56.7%. Discriminatory power did not statistically differ (*p* = 0.05) among markers or marker combinations. When species identification failed, the number of conflated species ranged from a mean of 4.3 (σ = 1.8) to 5.6 (σ = 4.1) species for individual markers and a mean of 2.9 (σ = 1.4) to 3.6 (σ = 2.1) species for marker combinations. There were no unambiguous statistical differences (*p* = 0.05) in the number of conflated species among markers or marker combinations.

A synergistic effect was observed for marker combinations both in terms of an increase in discriminatory power and decrease in the number of conflated species (Table 1; e.g. the two specimens of *Af. mannii* examined cannot be consistently distinguished from *Af. dawei*, *Af. falcatus*, *Af. gracilior*, or *Af. usambarensis* by any single marker, but when *matK* and nrITS2 are combined, *Af. mannii* can be consistently distinguished from all other species). In no case did combining markers result in a loss of discriminatory power.

The core barcode markers (*matK* and *rbcL*) were able to consistently distinguish among 46.3% of the species in the complete sample (Figure 3). In comparison, studies that analyzed sequences of *matK*, *rbcL*, and nrITS2, individually and in combination, using comparable methods of species discrimination (the ‘best match’ procedure [94] or the ‘simple pairwise matching’ technique [95]) had a median success rate of 59.5% (range  = 35.7–71.4) for core barcode markers (Table 2; [89], [90], [96]–[98]). In these same studies, species discrimination noticeably improved with the addition nrITS2 as a supplemental marker (median  = 92.6%; range  = 57.1–99.3). Although species discrimination did improve in Podocarpaceae with the addition of nrITS2 (Figure 3), the rate of species discrimination (56.7%) is less than the lowest published value (Table 2; [97]).

**Table 2:** Rates of discriminatory success for barcoding studies that analyzed *matK*, *rbcL*, and nrITS2 sequences using algorithms comparable to BRONX.

study focus	*matK*+*rbcL*	*matK*+*rbcL*+nrITS2
*Parnassia* (Parnassiaceae) [98]	71.42%	96.19%
Chinese *Primula* sect. *Proliferae* (Primulaceae) [96]	68.75%	87.50%
Malagasy *Euphorbia* (Euphorbiaceae) [90]	**59.45%**	99.32%
Caryoteae (Arecaceae) [89]	51.85%	**92.59%**
*Actaea* (Ranunculaceae) [97]	35.71%	57.14%

Median rates of discriminatory success are in boldface.

Of the 49 species represented by two or more individuals in the complete sample, BRONX could distinguish 28 (57.1%) from all other species using a combination of three markers (Table 1). In contrast, the ‘least inclusive clade’ method could distinguish 21 (42.9%) species (Figure 1). This provides another example of the poor performance of tree–based algorithms for barcode sequence discrimination [73], [74].

The complete sample was composed of 97 species represented by 90 distinct multilocus genotypes. Thus, if intraspecific variation is assumed to be near zero, one could plausibly expect that species discrimination would be close to 92.8%, however only 56.7% of species could be consistently distinguished using all three markers simultaneously.

In many cases, identification failed in spite of ostensibly useful variation being present—this most often occurred when genotypes were shared among species (e.g. *Po. guatemalensis* and *Po. matudae* are sister species [54] that have a total of three multilocus genotypes [Figure 1]: the first multilocus genotype is restricted to *Po. guatemalensis*, the second multilocus genotype is restricted to *Po. matudae*, and the third multilocus genotype is found in both species). In the cases where genotypes are shared across species boundaries, the data cannot definitively distinguish between the underlying causal mechanisms of recent introgression versus ancestral polymorphism followed by incomplete lineage sorting. In these cases, it is unlikely that sequence data from additional markers will increase species discrimination.

In some cases, identification failure is the result of an absence of sequence variation (e.g. *Dc. compactus* and *Dc. expansus* are sister species [54] that have identical sequences for all three markers [Figure 1]). Sequence data from additional markers may improve species discrimination in these cases. Although we did not test the utility of supplementary plastid markers, it seems unlikely that better discrimination will be provided by additional plastid data given the small difference in species discrimination between *matK* and *rbcL* (4.1%; Figure 3)—discriminatory power for plastid markers usually plateaus at two markers [95]. Rather than sequencing more plastid markers, effort would be better invested in variable unlinked markers that are easily recovered from Podocarpaceae (e.g. *NEEDLY* intron 2 [54], [99]).

Discrimination success was mixed for the two CITES–listed Podocarpaceae species (Table 1): *Po. parlatorei* (CITES Appendix I) can be distinguished from all other species using nrITS2 (*matK* and *rbcL* cannot distinguish *Po. parlatorei* from *Po. sprucei*; *rbcL* cannot distinguish *Po. parlatorei* from *Po. transiens*); *Po. neriifolius* (CITES Appendix III) cannot be distinguished from *Po. thailandensis* using all three markers (using single markers, *Po. neriifolius* can also be conflated with *Po. archboldii*, *Po. assamica*, *Po. brassii*, *Po. crassigemmis*, *Po. drouynianus*, *Po. gibbsiae*, *Po. insularis*, *Po. ledermannii*, *Po. philippinensis*, *Po. polystachyus*, *Po. ramosii*, *Po. rubens*, and/or *Po. subtropicalis*). The herbal dietary supplement, *N. nagi* (Asian bayberry), cannot be distinguished from *N. formosensis* using all three markers (*matK* also cannot distinguish *N. nagi* from *N. motleyi*).

### Barcode gap

**Figure 4:**
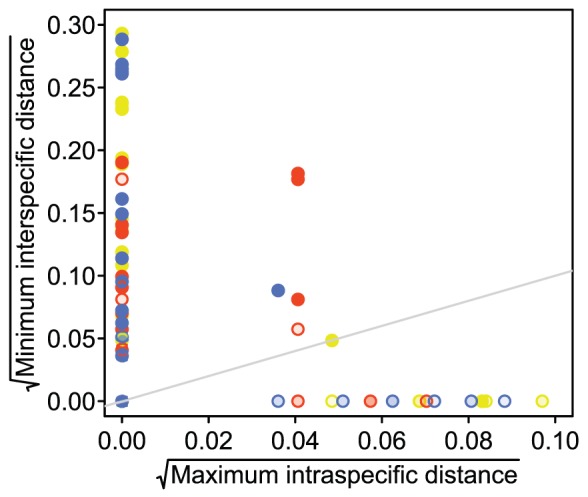
Barcode variation within and among species for complete samples. Circles represent the set of *matK* (blue), *rbcL* (red), and nrITS2 (yellow) sequences for each species. Opaque filled circles denote diagnostic sequence sets. Non–diagnostic sequence sets are indicated with semi–transparent filled circles. Equal intra– and inter–specific variation is marked by the gray line. Points above the gray line indicate species with ‘barcode gaps’.

The barcode gap is a measure of the relative variation within and among species [78]. In the complete sample, 39.1% of species had a barcode gap for *matK*, 34.0% for *rbcL*, 38.1% for nrITS2, and 50.5% for all markers simultaneously (Figure 4; Table 1). There is no statistical difference (*p* = 0.05) in the frequency of barcode gaps among markers. The presence of a barcode gap is not correlated with sample size in Podocarpaceae (*r_pb_* = 0.06; *p* = 0.27).

Barcode gaps quantify species distinctness at the barcode locus and thereby provide a crude measure of identification reliability (i.e. a species without a barcode gap may be more likely to be misidentified since it is not particularly distinctive; [71]). In this data set, whether a species can be consistently distinguished from all other species is unrelated to the presence or absence of a barcode gap (*p* = 0.02). For *matK* and *rbcL*, all of the species that can be consistently diagnosed have a barcode gap, but there are six species with barcode gaps that cannot be consistently differentiated from all other species (*matK*: *Dd. beccarii*, *Po. bracteatus*, *Po. novae–caledoniae*, *Po. nubigenus*, *Po. rumphii*, and *R. minus*; *rbcL*: *Manoao colensoi*, *N. wallichiana*, *Po. bracteatus*, *Po. pilgeri*, *Po. spinolosus*, and *Pr. andina*). In contrast, there are four species that do not have nrITS2 barcode gaps, but can be consistently diagnosed with nrITS2 (*Af. gracilior*, *Dc. imbricatus*, *Po. lambertii*, and *Pr. ferruginoides*). There are also four species that have nrITS2 barcode gaps that cannot be consistently differentiated from all other species using nrITS2 (*Po. bracteatus*, *Po. celatus*, *Po. longifoliolatus*, and *Po. sprucei*). There are no species with multilocus barcode gaps that cannot be consistently diagnosed using all three markers simultaneously, but there are six species that do not have multilocus barcode gaps that can be consistently diagnosed (*Af. gracilior*, *Af. mannii*, *Dc. imbricatus*, *Dc. kinabaluensis*, *Po. polystachyus*, and *Pr. ferruginoides*; Table 1).

The absence of a barcode gap coupled with discrimination success serves to contrast algorithmic approaches that use diagnostic nucleotide positions (i.e. those positions that consistently distinguish one species from all others) with distance–based methods. The presence of a barcode gap, does not guarantee that a species will be distinct. For example, a species may have a large amount of intraspecific variation combined with a small, but consistent, amount of interspecific variation rendering the species without a barcode gap, but consistently diagnosable—one nucleotide difference that consistently differentiates the species in question from all other species is all that is required. Thus, the absence of a barcode gap is a poor predictor of discrimination success.

The presence of a barcode gap coupled with discrimination failure is an artifact of the analysis conducted: barcode gaps were computed using only sequences in the complete sample whereas discrimination was calculated with a reference database composed of all sequences. Thus, the barcode gap calculation did not necessarily include samples with zero interspecific distance that were included in the discrimination calculation. Restricting the discrimination calculation to sequences in the complete sample would have overestimated discrimination success for Podocarpaceae. At the same time, calculating the barcode gap using all sequences would have resulted incomparable values.

Sampling of additional individuals cannot decrease the maximum intraspecific distance, nor can it increase the minimum interspecific distance. Thus, new sequence data for *matK*, *rbcL*, and nrITS2 will either maintain or decrease the number of species with barcode gaps. Likewise, the rate of species discrimination cannot improve, and will most likely deteriorate, with additional sampling of individuals. New sequences of unlinked markers may however increase the number of species with barcode gaps and/or improve the rate of species discrimination.

### Conclusions

The vast majority of barcode sequences generated for this study were of high quality (Figure 2). Even the lowest quality sequences exceeded the minimum requirements of the BARCODE data standard. In the few instances that low quality sequences were generated, the responsible mechanism could not be discerned: slip–strand mispairing at the site of repetitive sequence elements cannot adequately explain the low quality sequences observed.

The power of *matK*, *rbcL*, and nrITS2, individually and in combination, to discriminate among Podocarpaceae species is relatively low (56.7% of species at maximum; Table 1; Figure 3). There were no statistically significant differences in the discriminatory power of markers or marker combinations. Although the discrimination rate for Podocarpaceae is below the rate reported for comparably analyzed studies (Table 2), it is not markedly lower. Plant DNA barcoding studies that heavily sample within taxonomic groups usually report low rates of species discrimination.

Discrimination success was mixed for Podocarpaceae species important in commerce and of conservation concern (Table 1). The CITES Appendix I species, *Po. parlatorei*, can be distinguished from all other species using nrITS2. Unfortunately, the CITES Appendix III species, *Po. neriifolius*, and the herbal dietary supplement, *N. nagi*, cannot be unambiguously distinguished from all other Podocarpaceae using all three markers.

The presence of a barcode gap was not predictive of discrimination success. There was no statistically significant difference in the frequency of barcode gaps among markers in Podocarpaceae (Figure 4). In addition, there was no correlation between number of individuals sampled per species and the presence of a barcode gap.

Sequences of additional variable unlinked markers that are easily recovered from Podocarpaceae (e.g. *NEEDLY* intron 2) may increase the rate of species discrimination.

## Supporting Information

Dataset S1 Vouchers and GenBank accessions for samples of Podocarpaceae.
(TAB)
